# The Encyclopedia of Life v2: Providing Global Access to Knowledge About Life on Earth

**DOI:** 10.3897/BDJ.2.e1079

**Published:** 2014-04-29

**Authors:** Cynthia S. Parr, Nathan Wilson, Patrick Leary, Katja S. Schulz, Kristen Lans, Lisa Walley, Jennifer A. Hammock, Anthony Goddard, Jeremy Rice, Marie Studer, Jeffrey T. G. Holmes, Robert J. Corrigan, Jr.

**Affiliations:** † National Museum of Natural History, Smithsonian Institution, Washington DC, United States of America; ‡ Marine Biological Laboratory, Woods Hole, MA, United States of America; § Smithsonian Institution, Washington, DC, United States of America; | Harvard University, Cambridge, MA, United States of America

## Abstract

The Encyclopedia of Life (EOL, http://eol.org) aims to provide unprecedented global access to a broad range of information about life on Earth. It currently contains 3.5 million distinct pages for taxa and provides content for 1.3 million of those pages. The content is primarily contributed by EOL content partners (providers) that have a more limited geographic, taxonomic or topical scope. EOL aggregates these data and automatically integrates them based on associated scientific names and other classification information. EOL also provides interfaces for curation and direct content addition. All materials in EOL are either in the public domain or licensed under a Creative Commons license. In addition to the web interface, EOL is also accessible through an Application Programming Interface.

In this paper, we review recent developments added for Version 2 of the web site and subsequent releases through Version 2.2, which have made EOL more engaging, personal, accessible and internationalizable. We outline the core features and technical architecture of the system. We summarize milestones achieved so far by EOL to present results of the current system implementation and establish benchmarks upon which to judge future improvements.

We have shown that it is possible to successfully integrate large amounts of descriptive biodiversity data from diverse sources into a robust, standards-based, dynamic, and scalable infrastructure. Increasing global participation and the emergence of EOL-powered applications demonstrate that EOL is becoming a significant resource for anyone interested in biological diversity.

## Introduction

Biodiversity science has produced hundreds, if not thousands, of isolated database resources (Chavan and Ingwersen 2009, Page 2009, Lapp et al. 2011) in addition to a markedly diverse landscape of journals (Krell 2002). Though a growing number of projects recognize the value of and help connect their resources to global hubs (e.g. Gaiji et al. 2013, Roskov et al. 2014, Smith et al. 2011) many do not (Parr et al. 2012). Moreover, the value of open access, management of information overload, and engagement of the public is increasingly recognised (Wilson 2003, Goddard et al. 2011, Wheeler et al. 2012, Patterson et al. 2014).

The Encyclopedia of Life (EOL, eol.org) is an online database aiming to document all life on Earth. Globally and taxonomically comprehensive, EOL serves descriptive information and media (images, videos, sounds, maps) about biological organisms. While the modern concept of EOL was proposed by E. O. Wilson (Wilson 2003), it leverages earlier efforts such as the Tree of Life Web project (Maddison et al. 2007) and All Species Foundation (Blackmore 2006). The Smithsonian Institution leads EOL’s international consortium, which includes academic, government, and non-governmental institutions (Encyclopedia of Life 2012). Harvard University and the Marine Biological Laboratory host the Learning + Education (L + E) and Bioinformatics working groups, respectively.

EOL’s focus on description and illustration complements several related global efforts. The Catalogue of Life Partnership (CoL, Roskov et al. 2014) focuses on a comprehensive hierarchy of biological names. Global Biodiversity Information Facility (GBIF, Gaiji et al. 2013), focuses on primary biodiversity data – information on museum specimens, field observations, and results from experiments. The International Nucleotide Sequence Database Collaboration (INSDC, Benson et al. 2013), focuses on molecular genetic data. Like these initiatives, EOL was conceived in response to increasing demands for biodiversity information from scientists, policy makers, educators, formal and informal learners, and the general public. EOL integrates content from many sources but accepts and serves only materials in the public domain or explicitly licensed for re-use under Creative Commons licenses. EOL shares all content it collects with other projects by making it available for download through an Application Programming Interface (API). All new software developed for the project is open source and is supported by an open source software stack.

The task of documenting all life is vast, perhaps too vast for the relatively small community of formally-trained biodiversity experts (Wilson 2003, Wheeler et al. 2012). EOL has therefore put a strong emphasis on providing a platform for the collaboration of those experts and biodiversity enthusiasts without formal training. EOL is a Content Curation Community (Rotman et al. 2012a) rather than a wiki, as it combines aggregation, direct contribution, and curation processes. The integrity of individual contributions is always preserved, and attribution to original sources as well as information about review status are prominently featured in both the human and the machine interfaces to the site.

EOL’s first phase established a basic content aggregation and curation infrastructure with the original website launching in 2008 (Schopf et al. 2008). The second phase made improvements to make EOL more engaging, personal, accessible, and internationalizable. Version 2 was released in September 2011.

In this paper, we review recent developments added for Version 2 and subsequent releases through Version 2.2. We outline the core features and technical architecture of the system. We summarize milestones achieved so far, both to present results of the system implementation and to establish baselines upon which to judge future improvements and comparisons with other systems. Finally, we discuss the significance of the Encyclopedia of Life to the landscape of biodiversity informatics.

## Project description

### Design description

**Page redesigns**

**Figure 1.**
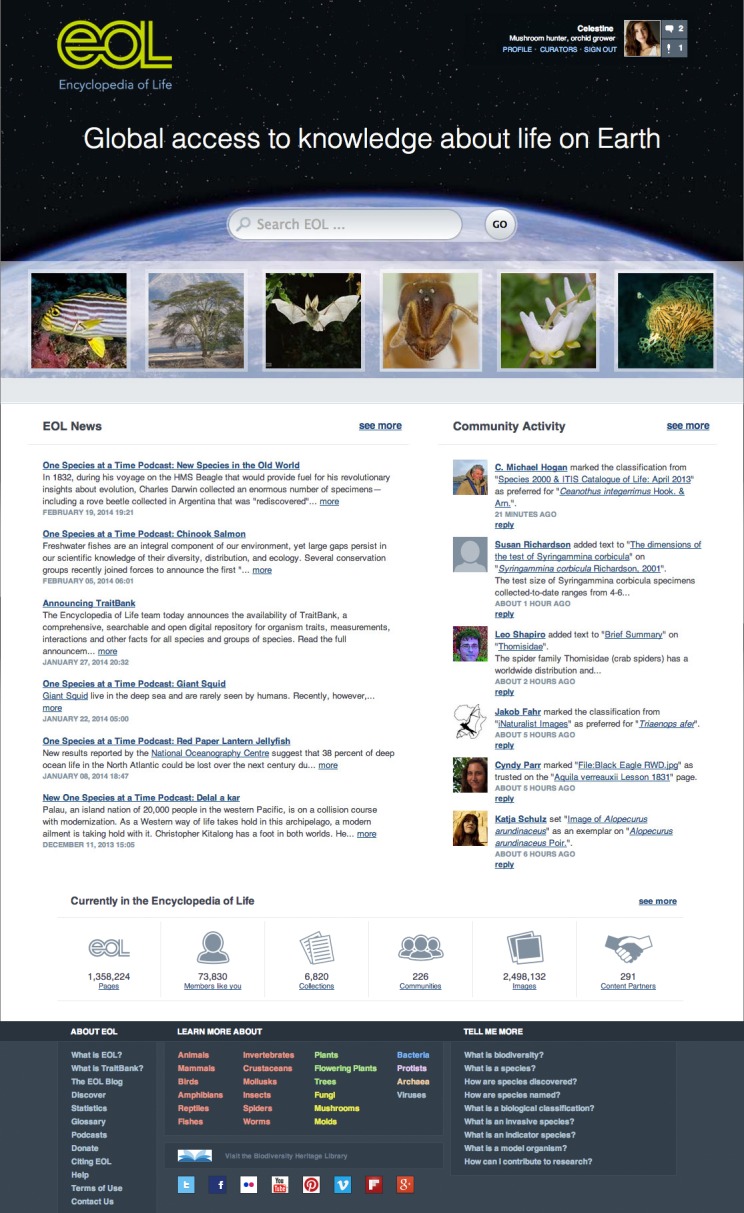
**EOL v2 Homepage.** When a member is logged in, the upper right corner of the page features links to member profile, personalized newsfeeds, and other information. Below the site search box, the "March of Life" thumbnails provide links to a sample of taxon pages drawn at random from pages above a minimal richness threshold. Two columns then feature EOL-related news items and an overview of recent community activity, followed by selected site statistics with a link to more detailed statistics over time. The site footer provides quick access to gateway pages (see below) and other site documentation.

**Figure 2.**
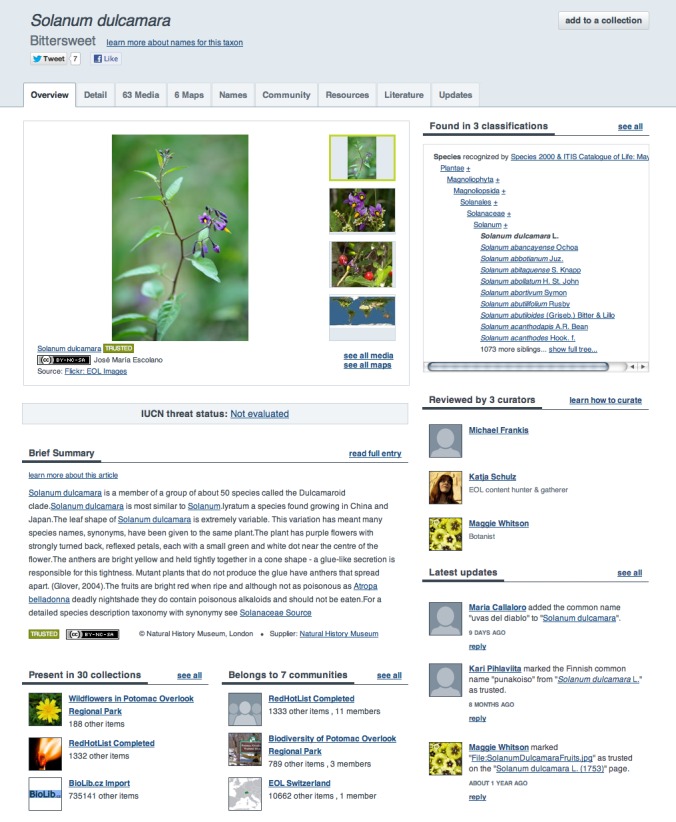
**The Overview tab** is the default view of an EOL taxon page. It features a sample of images, including a map, if available, a taxon hierarchy with links to other pages, a brief introduction to the taxon (if available), an activity feed, and links to relevant collections and communities.

EOL Version 2 involved a complete redesign of page styles to be more personal and engaging. In addition to the “March of Life” (a changing set of images linked to selected EOL pages), the homepage (http://eol.org, Fig. 1) now features recent activity from the site, better navigational links, and a more prominent search box. “Taxon Pages,” which provide access to all the information associated with a particular biological taxon (species, genus, family etc.) were completely revised to follow a tabbed paradigm. The default tab is an Overview that shows a sample of the kinds of information available on other tabs (Fig. 2). The Overview highlights a brief text summary (where available) for each taxon as well as a sample of multimedia: a classification, a map, recent discussions, and a few of the EOL **Communities and collections** that include the taxon (see below). The Overview tab becomes especially important as some pages accumulate not only rich content but also activity by users and curators.

**Comments and newsfeeds**

Commenting by users was available in the first version of EOL, but it has become a more central feature in EOL Version 2. Comments are now displayed much more prominently and are incorporated into EOL Newsfeeds, which also aggregate user actions relevant to the topic of the newsfeed. Newsfeed topics include users, taxa, collections and communities. EOL members (users who register for accounts on the site) are notified of responses to their comments and actions, and email notifications from newsfeeds can be customized in a preferences panel. The new EOL commenting system resulted in a roughly 4-fold increase in the rate of commenting compared to Version 1.

**Text and link contributions**

With the addition of a WYSIWYG editor to the existing text contribution interface, the authoring of taxon descriptions in the EOL interface has become easier in Version 2, and over 7,000 articles have been contributed in this way. In addition, we have introduced a link object so that contributors can submit well-described links to external resources; these are found on the Resources tab.

**Communities and collections**

EOL Version 2 introduced the ability for members to form communities and build collections (of taxa, of image objects, of other collections, etc.) on EOL, as described more fully below (Implementation). EOL collections allow users to collaborate on projects and to annotate and arrange EOL content from a personal point of view. Since the content of collections is available through the EOL API (see **Application Programming Interface** section below), they can be employed to organize EOL content for use by other applications. This collection-making facility likely is the most powerful new EOL feature; users can add value to the content by organizing it, and software developers can build on this value.

**Hotlists**

Most of the 1.9 million species described by science (Chapman 2009) are completely unknown to all but a handful of specialists. While it is important that these organisms be adequately represented in EOL, content development efforts focus on taxa that are of highest interest to EOL target audiences. To inform this content strategy, we compiled a series of taxon collections (“hotlists”) that cover commercially valued species (food, medicine, materials), rare and endangered species, invasive species, parasites and diseases, model organisms, and charismatic species. Content progress is continually assessed for the comprehensive hotlist comprising more than 90,000 taxa, and work with content partners is prioritized, in part, based on their potential to enrich pages of hotlist taxa.

**Presentation layer redesign**

The EOL Version 2 redesign included a complete rewrite of EOL’s presentation layer with the goal of delivering content in meaningful ways to the widest possible audience regardless of the recipient's device, ability or location. The structure, style and client-side behavior components of each page were separated and rewritten using progressive enhancement techniques (Champeon 2003). We adhered to best practices for document structure semantics (e.g. <h1> for page titles) to improve compatibility across devices such as screen readers and to lay the foundation for accessibility, search engine optimization, and internationalization.

**Accessibility and search engine optimization**

Design and architectural changes meet the World Wide Web Consortium (W3C) recommended Web Content Accessibility Guidelines (WCAG) 2.0 (Termens et al. 2008). While some adjustments were made purely for the benefit of accessibility, such as an extension of the EOL color scheme to include better color contrast ratios, the majority of accessibility improvements were carried out in conjunction with search engine optimization due to overlaps between the requirements of screen readers and web crawlers. A Sitemap was generated to instruct search engines which pages are recommended to be indexed. The sitemap was intentionally kept small and designed to feature the most useful pages to maximize the benefit of search engine crawlers. For example, links to Taxon Overview pages are included in the sitemap, but not links to other Taxon Page tabs. About 4 million pages are included in the sitemap (instead of more than 60 million if all tabs were included). This work has had the concrete result of raising the overall Google PageRank of the site (Page et al. 1998).

**Internationalization and localization**

In partnership with Bibliotheca Alexandrina, the EOL interface system (menus, controls, feedback messages, etc.) was fully internationalized. This work, combined with the separation of structure and style, has allowed the site to support the right to left layout needed for some non-Latin languages such as Arabic. Translation of site elements into specific languages was often accomplished by EOL global partners, e.g. Spanish by Costa Rica's Instituto Nacional de Biodiversidad and Simplified Chinese by the Chinese Academy of Sciences. The abstraction of interface strings has also enabled a partnership with the TranslateWiki platform, which supports interface translation by volunteers into over 120 languages. Once a language reaches a translation threshold of 75%, it is added to the menu of supported languages on EOL. This process has resulted in support of 16 languages in addition to English, with active development continuing for several more. EOL currently displays only text object content that matches a user's preferred language setting, but provides links to content available in other languages. Following these links changes the language setting. The goal is to avoid rendering multiple languages on the same page.

**Gateway pages and podcasts**

To better support beginning users, EOL now provides pages on general topics such as “What is biodiversity?” and introductory pages to major groups of organisms. Some of these pages are adapted from partner projects such as the Encyclopedia of Earth or the Animal Diversity Web (Myers et al. 2013). These pages now appear in the footer on every EOL page. They provide orientation to a topic and suggest internal and external links. In addition, the Harvard Museum of Comparative Zoology (http://www.mcz.harvard.edu/) sponsors the EOL *One Species at a Time* podcast series. These are five-minute CC-BY licensed stories for classroom learning hosted at http://podcast.eol.org/podcast and appearing on relevant EOL pages.

### Funding

Support was provided by John D. and Catherine T. MacArthur Foundation (93466-0 amendment to grant 06-89123-000-GEN), Alfred P. Sloan Foundation (2009-6-076), Smithsonian Institution, Marine Biological Laboratory, and Harvard University.

## Web location (URIs)

Homepage: http://eol.org

Wiki: http://wiki.eol.org

Blog: http://blog.eol.org

## Technical specification

Programming language: Ruby on Rails, PHP

Service endpoint: http://eol.org/api

## Repository

Type: Git

## Usage rights

### Use license

Other

### IP rights notes

Third-party content copyright remains with rightsholders. All content is either in the public domain or licensed for re-use with Creative Commons licenses. All but non-derivative ND licenses are accepted for third-party content (see EOL Policy). User-generated content (e.g. comments, annotations in collections) is CC-BY licensed according to the Community Conditions and Comment policy. All EOL-generated source code is available under the MIT License.

## Implementation

### Implements specification

**Core system features**

**Figure 3.**
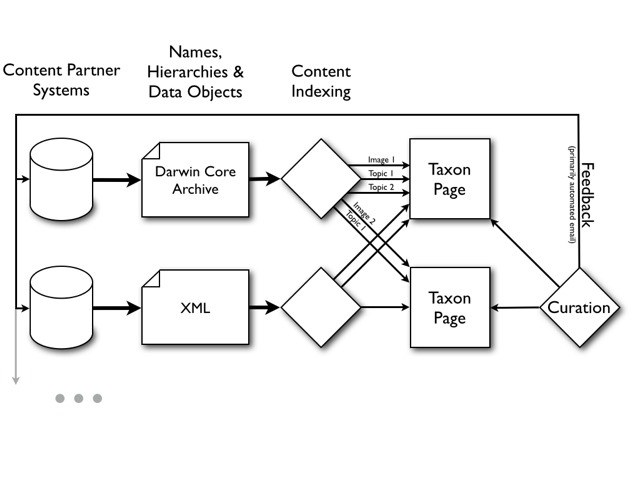
**High-level data flow from content partners into EOL.** Content partners make available EOL data transfer documents (resource documents) that are periodically indexed by EOL. These provide names, name hierarchies, and associated data objects to EOL. EOL aggregates these data and presents them on Taxon Pages. The content assigned to a Taxon Page can be reviewed, hidden, or reassigned to other Taxon Pages by EOL Curators.

Fig. 3 provides a conceptual overview of how most information on EOL is assembled.

**Names infrastructure**

Resource documents made available by content partners define the text and multimedia being provided as well as the taxa to which the content refers, the associations between content and taxa, and the associations among taxa (i.e. taxonomies). Expert taxonomists often disagree about the best classification for a given group of organisms, and there is no universal taxonomy for partners to adhere to (Patterson et al. 2008, Rotman et al. 2012a, Yoon and Rose 2001). As an aggregator, EOL accepts all taxonomic viewpoints from partners and attempts to assign them to existing Taxon Pages, or create new Taxon Pages when necessary. A reconciliation algorithm uses incoming taxon information, previously indexed data, and assertions from our curators to determine the best aggregation strategy.

This taxonomic reconciliation process involves comparing the preferred scientific names, synonymy, and taxonomy from an incoming resource document to the same information from all previously indexed resources. It is designed to merge taxa based on synonymy (for example when the preferred name of one taxon is in the synonymy of another) and keep taxa separated that are homonyms (the same scientific name appearing in two distinctly different clades like *Morus* which is a genus of both birds and plants). Rank information is important to the reconciliation process as it permits the differentiation of cross-rank homonyms. For example, there is a genus of seaweed known as *Vertebrata* and the same name is used for the group of all organisms with backbones. Reconciliation is an automated process and can make incorrect decisions, so there is a series of operations EOL curators can perform to manually resolve taxonomic and typographic inconsistencies. Ultimately, multiple taxonomic views indexed by EOL are displayed in the Names Tab of a Taxon Page, and EOL curators can choose a preferred taxonomy to display for browsing on the Overview tab.

Partners can provide common names and synonyms as part of their taxon definitions. Synonyms are used by EOL to help determine which taxon definitions should be aggregated into the same Taxon Pages. They are also valuable search keywords that help users find the pages they are looking for.

Previous studies suggest that common names are often more valuable for search than scientific names or synonyms (Parr et al. 2004). Common names vary by language and region and as such are an important component of an internationalized EOL. As a user changes their browsing language, common names shown throughout the site will change to match the user’s preferred language. Curators have control over which common names are shown as preferred for each taxon in each language, and can add new common names when needed.

**Taxon pages**

Taxon Pages are the main organizational unit of EOL, presenting a standardized page for every taxonomic entity that the system recognizes. Each Taxon Page has 9 tabs: Overview, Details, Media, Maps, Names, Community, Resources, Literature, and Updates, plus an additional tab for EOL curators, Worklist. The default tab, Overview (Fig. 2), offers a sample of information available on the other tabs and links to more detailed information. The Details, Media, Maps, Names, Resources, and Literature tabs display aggregated, topical information about the taxon as provided by EOL partners and members, including interactive GBIF occurrence maps and references from the Biodiversity Heritage Library, BHL (Norton 2008). BHL runs TaxonFinder on its documents to find biological name strings (Wei et al. 2010), which EOL then indexes. Any time the BHL tab is visited on a Taxon Page the system offers links to all pages in BHL that include any of the scientific (not common) names of the taxon page.

The Community tab offers information about what EOL Communities and Collections are interested in the taxon, and who the curators of the taxon have been. The Updates tab lists all of the comments on the Taxon Page as well as statistics about the content on the page, including the page’s Richness Score (see **Richness score** below).

**Data objects**

Images, text, videos, sound files, and maps provided by content providers and EOL members are referred to as “Data Objects”. Data Objects are the building blocks of EOL. Taxon Pages are populated through the aggregation of relevant Data Objects from multiple sources. Each Data Object also has its own dedicated page that contains information about the taxon (or taxa) the Data Object is associated with, license information, all available source and attribution information, a tool for rating the Data Object, links to other versions of the Data Object, comments on the Data Object, and, for non-text objects, a text description (caption) if available. These Data Object Pages are accessible through links from EOL Taxon Pages and through their own unique URLs (e.g. http://eol.org/data_objects/21942847). EOL curators have access to tools on the Data Object Page to control visibility and trusted status, and on image Data Object Pages, tools to crop images to create versions of thumbnail images that are shown throughout EOL. While curators can hide a Data Object or indicate its trusted status, the content itself can only be changed or updated by the provider.

**Darwin Core Archive support for content ingestion**

Initially, EOL harvested resource documents formatted according to an XML transfer schema drawing from standards such as Dublin Core, Darwin Core (Wieczorek et al. 2012), and TDWG Species Profile Model (SPM). We also accepted Excel spreadsheets based on a template incorporating these standards. Beginning in May 2012, EOL began accepting datasets formatted as Darwin Core Archives (DwC-A), a biodiversity informatics community standard (Baker et al. 2014). For details of the EOL implementation, see http://eol.org/info/cp_archives. XML datasets continue to be supported, but we recommend that all new partners provide DwC-A. Darwin Core Archives are very extensible and flexible, with a meta.xml file providing information about the elements included in flat tabular files and instructions on how machines should read them. Providers can design their DwC-A to suit more than one consumer or to adhere to content standards such as Plinian Core. They are readily understandable by scientists more comfortable with tabular formats; EOL’s new spreadsheet template is very similar to a formal DwC-A.

**Building the content**

Most EOL content is aggregated via content partner tools (designed for projects that have large amounts of content to share) or added directly to the web site by users. Any EOL member can add and manage an EOL content partner account through their member profile (see http://www.eol.org/cp_getting_started). After supplying basic information about their project, users register one or more resource documents that contain the information they want to share. Resource documents may be customized exports from a database, they may be created by programs that parse web pages or call web services, or they may be manually assembled spreadsheets. Some resources are the result of newly published marked-up taxonomic treatments (Miller et al. 2012, Penev et al. 2010) while others are taxonomic treatments from legacy literature (Plazi.org). A resource can be a checklist of taxon names, or it can be a classification with or without references. Most resource documents include text objects or point to multimedia objects and provide their associated metadata. EOL staff members are available to assist in preparing resource documents and must approve the first publication of the resource on EOL. After that, content may be harvested and automatically published on a schedule in order to maintain synchrony with source databases. Each resource that is harvested generates an automatically updating EOL collection (see **Communities and collections** below) as well as a panel of traffic statistics that are made available to partners.

Currently, EOL members can add text objects, also known as articles, directly to EOL using the “Add an Article” button on the Details tab. Multimedia objects cannot be uploaded directly to EOL but must be added through partners such as Flickr, Wikimedia Commons, iNaturalist, Vimeo, YouTube, and Soundcloud.

**Richness score**

**Figure 4.**
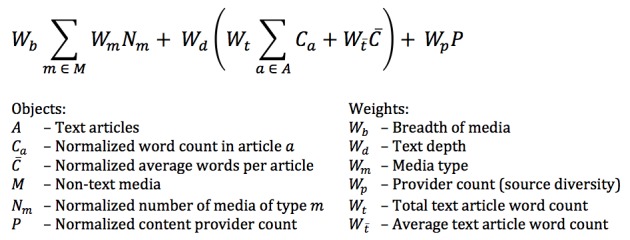
**The EOL richness score** is the sum of three weighted components: breadth, depth, and diversity. Breadth considers the different media types of information objects (including the number of different subjects available for text), depth considers both average and total number of words in text objects, and diversity considers the number of different sources of information, or providers. Normalized object values are scaled to be between 0 and 1 and put on a log-based scale such that the first objects counts more than the second up to a chosen limit at which point the value is 1 and additional objects of that type have no impact on the richness. The final score is multiplied by 100, so that it ranges from 0 to 100. For more detailed information, see http://eol.org/info/richness_score.

EOL has developed a Richness Score for taxon pages (Fig. 4) that is inspired by community ecology’s diversity indices (Peet 1974 among others) which are rooted in information theory. Ranging from 0-100, the Richness Score combines information on the number and review status of text and multimedia objects, the number of words and distinct topics of text objects, and the diversity of sources. These factors are assigned weights and limits (having 200 images may not make a page much more rich than having 25 images). To develop the richness algorithm, we sampled dozens of pages and had staff assess them for their gestalt “richness” based on their own criteria. Then we compared those scores to scores generated by the algorithm, and iteratively changed weights until we achieved a set of weights that appeared to reflect human perception of “richness.” The algorithm may be occasionally adjusted based on user input. The Richness Score and its components are listed in the Updates tab of each Taxon Page and is also available through the API. It is used to prioritize pages for display in search results, API calls, and the rotating ‘March of Life’ slideshow on the EOL home page. A page with a score of 40 is currently considered "rich."

**Communities and collections**

EOL Communities provide a way to group users. The primary value of this feature at the moment is to share the management of different EOL Collections. They also provide a simple forum through the associated newsfeed. Collections provide a way for users to organize, annotate, and share the content on the site. Collections may range from species lists for local areas (e.g. Florida Native Plants) to lists of homonyms (Homonyms on EOL) to content collections for education or entertainment (e.g., X-ray Vision: Fish Inside Out). Many different types of items within EOL can be collected including Taxon, Image, Article, User, Community and even other Collection pages (e.g., a collection of video collections). Collections can be viewed as a visual gallery, a simple list, or an annotated list and can be sorted in a variety of ways including by Richness Score. Annotation fields allow Collection managers to provide notes, references, or sort fields for each item in the collection. By default, an EOL Collection is managed by the user who creates it. However, any manager can share management privileges with other EOL members or communities.

**Curation**

EOL provides curation tools for volunteer data curators. All curators must register under their real names. To facilitate participation of EOL members with different levels of expertise, three different curator levels are distinguished. As of April 2014, almost 300 EOL members have registered as assistant curators and over 1,300 members have been approved as full or master curators.

The Assistant Curator status requires no qualifications and conveys limited curation powers. Assistant Curators can add taxon associations to data objects (e.g., to identify organisms shown in an image), but these associations are marked as "unreviewed" until confirmed by a Full Curator. Assistant Curators can also add common names, select preferred common names, select exemplar images and articles, and crop image thumbnails. They are encouraged to add text and help find problems that Full Curators can resolve. Full Curators must have credentials (e.g. relevant professional affiliations, publications, membership in a professional association). In addition to the powers of Assistant Curators, they can trust or untrust text or multimedia objects and select preferred classifications for taxon pages. Master Curators can manage taxon concepts (overriding the automated reconciliation process by merging or splitting classifications featured on a given taxon page) and delete comments that do not adhere to EOL community policies.

Untrusted content is hidden from public view but still visible to Full and Master Curators for further review. Curation actions and comments are reported to content providers (Feedback, in Fig. 3) so that the problem can be resolved at the source. In the case of multiple curation actions on a single object, name or classification, the current review or priority status reflects the decision of the most recent curator. The display sequence of data objects on EOL pages is also affected by user ratings (on a 1 to 5 scale) which can be submitted by any EOL member. Object ratings are averaged across all raters, with ratings by curators carrying more weight. Curators can work directly on EOL taxon and data object pages, or they can use the EOL Worklist tab which provides an interface for to quickly find taxon-specific content that is unreviewed or recently added or to filter by particular providers like Wikipedia or Flickr.

**Search**

The EOL website search is configured to find scientific names and common names, with preference in search result ordering given to preferred scientific names (names that have been manually selected by curators as “preferred” for a taxon) first, followed by preferred common names, and synonym. EOL search also indexes Communities, Collections, EOL members, Data Objects, and EOL documentation pages, and search results can be filtered by these categories. If there is a best result, the system takes the user directly to that taxon page, with an option to return to the search results page to view other results.

**API**

The EOL Application Programming Interface (API) allows content indexed by EOL to be easily accessible to other websites and software developers. Through the API, applications can search EOL Taxon Pages, fetch page metadata such as names, images and text, and access hierarchy and collection information. The latest versions of API methods allow data to be returned in either XML format or the simpler JSON format. Method documentation has been improved and internationalized, and now includes forms to help users test the methods and their various parameters by interactively showing the responses. An example of a website using the EOL API to feature EOL data within their own site is the Smithsonian National Museum of Natural History’s Species of the Day widget. This widget is created using the API to draw data from a custom EOL Collection. Other examples include various games, visualizations, and other sites that re-use EOL content.

**Technical architecture**

**Figure 5.**
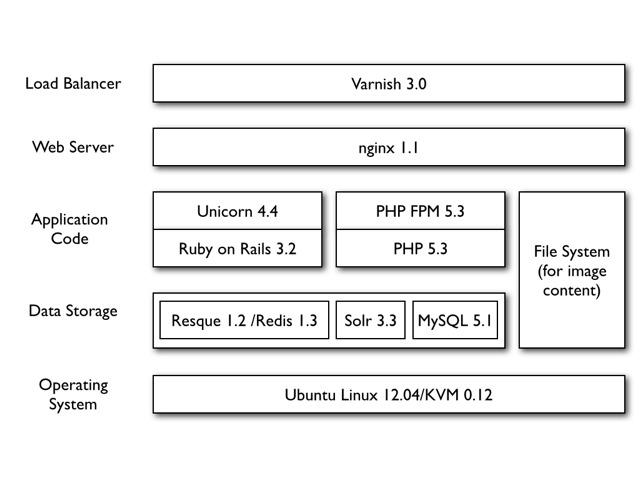
**Software stack for the production EOL web-servers.** The vast majority of the code written specifically for EOL is in Ruby – which handles the website and the API – and PHP, which handles the content import process and provides some administrative interfaces.

EOL Version 2 provided an opportunity to significantly improve the hardware and software infrastructure of EOL. The entire software and hardware stack supporting the serving of eol.org moved to the Research Computing group at Harvard University while remaining managed by the EOL Operations team at the Marine Biological Laboratory. The new architecture introduced KVM-based virtual machines to the infrastructure, allowing a more efficient use of resources and faster deployment of new infrastructure services to support the hosting of the site (Fig. 5). The open source tools Chef and Capistrano were used to create a new mechanism for deploying the application based on data stored in Github. Resque is used to managed the email notification and download queues.

The EOL technical team uses a modified version of the Scrum software development framework (Schwaber 2004) to plan, develop, and improve EOL features. More information about the specific approach that the EOL team uses is available at http://eol.org/info/development.

### Audience

EOL has a worldwide audience including experts, enthusiasts and casual visitors. About 39% of user sessions originate in the United States and more than 47% of user sessions originate in countries where English is not an official language. Starting with v2, visitors registering to become EOL members were invited to select one or more audience categories to describe themselves. Of 6,410 people who self-identified by 18 April 2014, 47% chose "enthusiast", 36% chose "student", 20% chose "educator", 18% chose "citizen scientist", and 20% chose "professional scientist". However, this distribution may not reflect the more than 73,000 current EOL members or the vastly larger number of visitors who never register or who encounter EOL content primarily via social media channels.

Experts and enthusiasts are encouraged to participate in EOL as content curators. As of April 2014, almost 300 EOL members have registered as assistant curators and over 1,300 members have been approved as full or master curators.

At least in North America, the formal education audience is an important demographic for EOL. We see from Google Analytics that there are increases in the use of the site when most schools are in session. The EOL Learning & Education group also actively posts information on about 15 listservs, including the National Science Teachers Association (NSTA), Scuttlebutt (NOAA Marine Education site) and the Ecological Society of America's EcoLogic Listserv.

## Additional information

### Milestones

**Figure 6.**
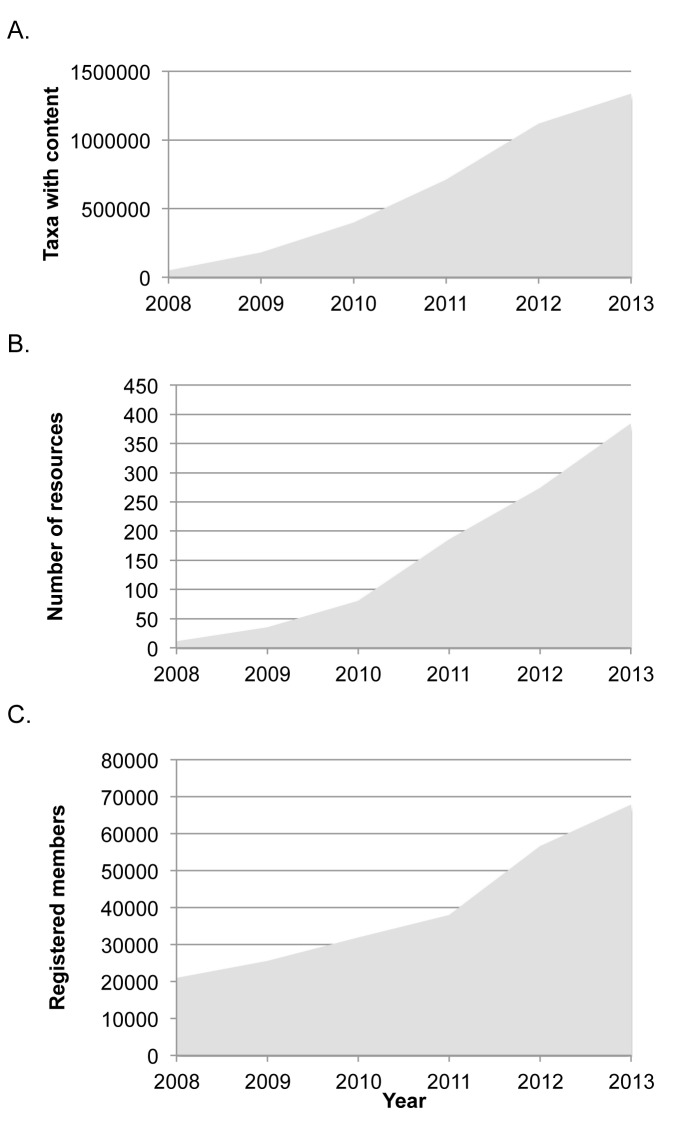
**Growth in Encyclopedia of Life from 2008-2013.** **A.** Taxon pages with content (at least one text article, image, map, video, or sound) (Suppl. material 1). **B.** Published resources (content import files). A provider may submit more than one resource file, for example when providing different kinds of content (Suppl. material 2). **C.** Registered EOL members (Suppl. material 3).

EOL’s growth in overall information, provider resources, and membership has steadily increased (Fig. 6). EOL launched in 2008 with information on approximately 40,000 taxa. In 2012, EOL passed a significant milestone: more than 1 million pages had at least some text or multimedia content. Based on data through July 31, 2013, these pages now contain more than 3,192,609 text articles and 1,812,295 image objects, all showing explicit expert review status (especially important for content from large crowd-sourced partners such as Wikipedia or Flickr). About 112,000 pages have a Richness score of 40 or higher (out of 100), with 50% of the 90,000 hotlist pages meeting this threshold.

Still, most EOL pages remain without content, i.e., EOL provides nothing but a taxon name, and in some cases author information and a reference. Overall, EOL has indexed about 3.5 million taxa. This represents most of the 1.9 million extant (Chapman 2009) and 250,000 fossil species (Prothero 2013) described by scientists, as well as higher taxa (genera, families, orders, etc.), infraspecific taxa (subspecies, varieties, etc.) and hybrids (mostly in plants and some vertebrates), taxa whose names will eventually turn out to be synonyms (Alroy 2002, Joppa et al. 2011, Stork 1997), and more than 700,000 provisionally named taxa from molecular genetic data sources like the National Center for Biotechnology Information (NCBI) and Barcode of Life (BOLD).

**Figure 7.**
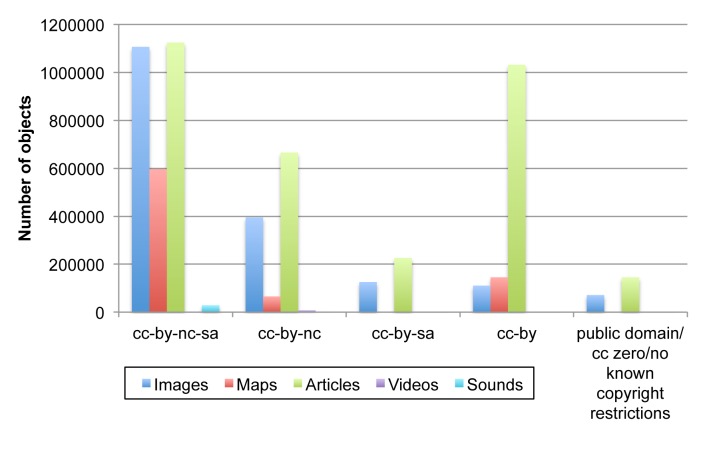
**Distribution of Creative Commons and other licenses for data objects on EOL**. CC-BY = Creative Commons Attribution license; NC = Non-commercial restriction; SA = Share-alike restrictions. Objects with gnu-gpl/gnu-fdl licenses (3903 images and 21 text articles) are not shown. Overall, as of July 2013, EOL has 3,192,609 text articles, 1,812,295 images, 806,664 maps, 30,366 sounds, and 10,219 videos (Suppl. material 4).

Closer examination indicates that EOL has an uneven distribution of content across languages, licenses, and topics. While EOL has vernacular names in 163 languages (Table 1), it has text objects in only 17, with the vast majority (97%) still in English (Table 2). While a significant amount of text content is shared under open licenses as defined by http://opendefinition.org (44%, public domain, CC-BY, CC-BY-SA), providers of multimedia content still prefer the more restrictive licenses that EOL permits (Fig. 7). The most frequent topic of EOL text articles (objects) is “Distribution.” Combined topics that cover multiple subjects, such as brief summaries and comprehensive descriptions, are also frequent (Table 3).

To date, users have created more than 5,000 EOL Collections. Many collections (approximately 35%) are for specific geographic regions and represent user-generated checklists that could be useful for refining map queries in areas where occurrence data are not yet available. Presence of a taxon or object in many user-generated Collections could be used (by EOL or by others) to sort or filter search results so that they are most relevant to user needs. Collection statistics, along with traffic statistics, could also help researchers explore the factors that make an organism or data object more engaging to broad audiences.

**Figure 8.**
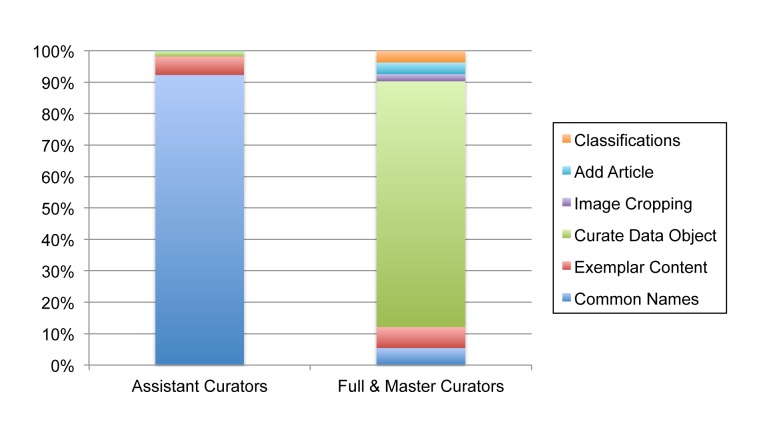
**Activity patterns of EOL Assistant Curators compared to Full and Master Curators.** Only Full and Master Curators can select preferred classifications and change the visibility and trust status of text and multimedia objects. Data Object curation by Assistant Curators is limited to adding associations between Data Objects and taxa.

**Figure 9.**
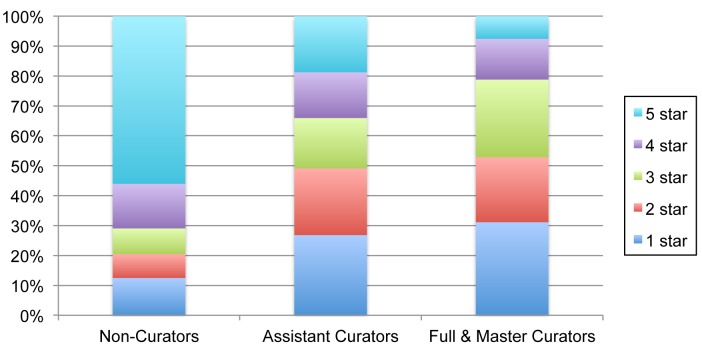
**Data Object rating patterns of EOL members in relation to their curator status.** Five stars is the highest rating, while one star is the lowest rating a member can give a text or multimedia object.

Though there is room for growth in curation activity, EOL is increasingly in a position to improve data quality across its network of providers. In July 2013, EOL had 1,258 registered curators (250 Assistant, 1,001 Full, 7 Master) of which 163 have been active in the last 12 months. In comparison, iNaturalist has 94 curators and the World Register of Marine Species has 826 editors (a thoughtful analysis of curation power across projects with different models is beyond the scope of this paper). The majority of data objects are considered trusted (92%), most having been acquired from authoritative sources. An average of 905 objects per month are being curated. Assistant and Full Curators have different patterns of activity, not surprisingly given their different access to tools (Fig. 8). Assistant Curators have provided many non-English common names shown in Table 1. Full Curators tend to have more rigorous rating patterns than either Assistant Curators or non-curators (Fig. 9). A previous study found evidence that activity by curators increased commenting activity of non-curators (Ahn et al. 2012).

In the period from August 2012 through July 2013, EOL was visited by 3.7 million unique users. About 44% of visits are from North America (including Mexico). Thirteen countries on other continents contributed a significant number of visits.

### Discussion

**Figure 10.**
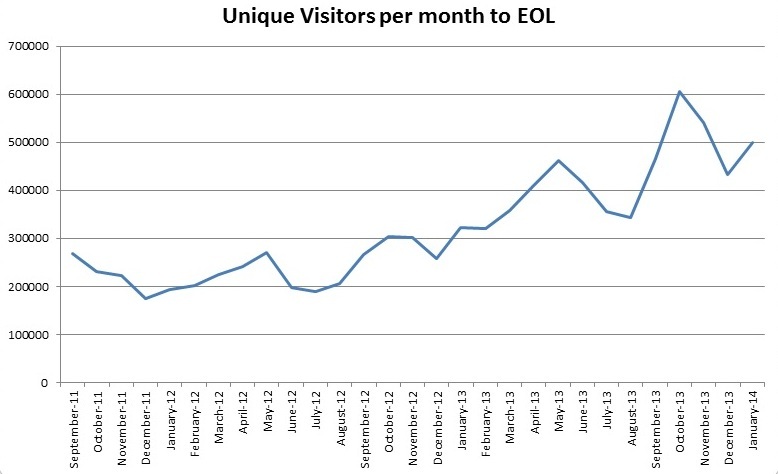
**Unique visitors to EOL per month**, September 2011-January 2014, per Google Analytics.

**Figure 11.**
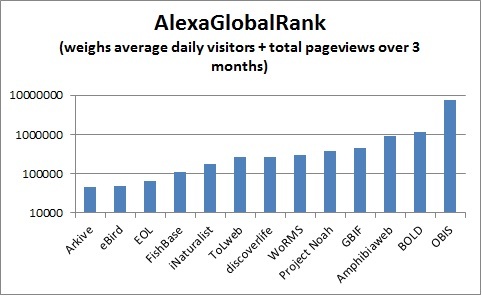
**Global Rank for biodiversity web sites** per http://www.alexa.com/, 02/26/2014. The rank is calculated using a combination of average daily visitors to this site and pageviews on this site over the past 3 months. Lower numbers indicate greater importance, as the site with the highest combination of visitors and pageviews is ranked #1. Note however that Alexa rankings are known to be subject to considerable sampling bias since they are largely based on the behavior of users browsing with an Alexa-compatible toolbar (Lo and Sharma Sedhain 2006).

**Figure 12.**
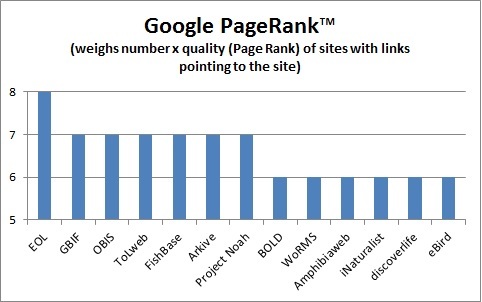
Google PageRank™ of various biodiversity websites, per http://www.prchecker.info/, 02/22/2014. Larger numbers indicate greater importance, and webpages with a higher PageRank are more likely to appear at the top of Google search results.

EOL has established its role of improving access to biodiversity information by aggregating and standardizing descriptive information and multimedia objects currently available across many otherwise isolated resources. It provides the infrastructure to connect both major hubs and independent projects (Parr et al. 2012). It is well positioned to provide connectivity and added visibility to partner projects. Visitor traffic to EOL has increased steadily since the launch of Version 2 (see Fig. 10) and has averaged 481,000 unique visitors/month over the past six months. It is natural that EOL should have higher traffic and visibility than either sites designed for a professional audience (WoRMS, GBIF, BOLD, OBIS) or sites designed for a less casual, more engaged audience (iNaturalist, Discoverlife, Project Noah) or sites that are more narrowly focused (Fishbase, ToLweb, AmphibiaWeb). This is reflected in the Global Rank of alexa.com, (which weighs traffic; Fig. 11) and in the Google Page Rank, (which weighs centrality, i.e., number and quality of links pointing toward a site; Fig. 12). Successful sites specializing in charismatic fauna (eBird, Arkive) can show higher traffic, but still have lower centrality. This is why linking back to content providers at every opportunity is so critical to EOL's mission; the role of a high visibility node is to connect high value, low visibility nodes to traffic that might not otherwise find them.

EOL complements long-term archives and metadata registries, e.g. DataONE (Michener et al. 2012) and Dryad (Vision 2010), by focusing on data mobilization – organizing and providing access for new users and new uses, while maintaining source provenance and rich attribution. It also plays an important role in aggregating images and type specimen information from museum collections, essentially leveraging their specimen-level digitation efforts for biological discovery and education. With a superset of taxa and selected information from all of its partners, EOL has more breadth than any of its largest sources (e.g. Gaiji et al. 2013, Costello et al. 2013, Benson et al. 2013). It is likely to be the richest single source of taxonomically indexed CC-licensed multimedia content about biodiversity. EOL's CC-licensing requirements have resulted in the application of such licenses to much content that might otherwise have remained All Rights Reserved (C. Parr personal observation) and has already fostered re-use of content by third party applications. It will be interesting to see if the usage of NC licenses, which many find to be problematic (Hagedorn et al. 2011) decreases or increases over time.

By taking a phased approach (phase 1 of core infrastructure and phase 2 of engagement), EOL has successfully built a professional, usable platform at a scale appropriate to its task of serving global biological information to multiple international audiences. Because it is scalable, as EOL grows, its Richness Scores can be used to assess the availability and quality of knowledge across the tree of life, especially when extended to structured data. The scores could also enable assessment of individual contributions and standardization (Liolios et al. 2012) and direct future investment in data capture and research.

Several challenges remain to be tackled in future phases. While there is some evidence (growth in collections, emergence of third party applications, curator activity, user traffic) of effective impact on and engagement by various audiences, tools for community and curator engagement are not as successful as hoped and so they may require more tailored experiences and effective feedback (Rotman et al. 2012a, Rotman et al. 2012b). EOL itself may be too large and diffuse to support effective communities. To satisfy the needs of the academic community, EOL must continue to seek better ways to provide professional, quantitative credit for the individuals and institutions who have curated or contributed content or functionality to the system (Liolios et al. 2012, McDade et al. 2011, Maddison et al. 2012) we also plan to enable phylogenetic views and access to associated data in collaboration with the Open Tree of Life project. While taxon names management has been automated to a large extent, more work is needed to reduce the need for manual curation and to better integrate EOL systems with relevant systems built by Global Names, i4Life, and iPlant (Boyle et al. 2013), to name a few. Some of the 3.5 million taxon pages will represent extinct taxa, subspecies or provisionally named taxa (many of these resulting from dark taxa shared by GenBank, Parr et al. 2012). Other taxa are likely to be useful for the Catalogue of Life, which has 1.4 million of the expected 1.9 million described species (Chapman 2009).

The next phase of EOL moves beyond the limits of encyclopedic text and multimedia to add the ability to ingest and serve highly structured data (numeric and controlled vocabulary terms with rich semantics) about the attributes of and relationships among organisms (Parr et al. in review). In the same way that EOL has helped to bring together and connect text and media from isolated sources, we aggregate structured data to provide a broad-scale view of analyzable biodiversity data. EOL’s standardized open access also facilitates new text mining or crowd-sourcing efforts to extract structured data about biological diversity, e.g. Thessen and Parr 2014).

## Supplementary Material

Supplementary material 1 Taxa with Content Data type: Comma-Separated-Values
Brief description: Taxon pages with content (at least one text article, image, map, video, or sound).
File: oo_6172.csv Cynthia Parr, Nathan Wilson, Patrick Leary, Katja S. Schulz, Kristen Lans, Lisa Walley, Jennifer A. Hammock, Anthony Goddard, Jeremy Rice, Marie Studer, Jeffrey T. G. Holmes, Robert J. Corrigan, Jr.

Supplementary material 2 Number of Resources Data type: Comma-Separated-Values
Brief description: Published resources (content import files). A provider may submit more than one resource file, for example when providing different kinds of content.
File: oo_6173.csv Cynthia Parr, Nathan Wilson, Patrick Leary, Katja S. Schulz, Kristen Lans, Lisa Walley, Jennifer A. Hammock, Anthony Goddard, Jeremy Rice, Marie Studer, Jeffrey T. G. Holmes, Robert J. Corrigan, Jr.

Supplementary material 3 Registered Members Data type: Comma-Separated-Values
Brief description: Registered EOL members.
File: oo_6174.csv Cynthia Parr, Nathan Wilson, Patrick Leary, Katja S. Schulz, Kristen Lans, Lisa Walley, Jennifer A. Hammock, Anthony Goddard, Jeremy Rice, Marie Studer, Jeffrey T. G. Holmes, Robert J. Corrigan, Jr.

Supplementary material 4 License Distribution Data type: Comma-Separated-Values
Brief description: Distribution of Creative Commons and other licenses for data objects on EOL.
File: oo_6175.csv Cynthia Parr, Nathan Wilson, Patrick Leary, Katja S. Schulz, Kristen Lans, Lisa Walley, Jennifer A. Hammock, Anthony Goddard, Jeremy Rice, Marie Studer, Jeffrey T. G. Holmes, Robert J. Corrigan, Jr.

Supplementary material 5 Curator Activity Data type: Comma-Separated-Values
Brief description: Activity patterns of EOL Assistant Curators compared to Full and Master Curators.
File: oo_6176.csv Cynthia Parr, Nathan Wilson, Patrick Leary, Katja S. Schulz, Kristen Lans, Lisa Walley, Jennifer A. Hammock, Anthony Goddard, Jeremy Rice, Marie Studer, Jeffrey T. G. Holmes, Robert J. Corrigan, Jr.

Supplementary material 6 Data Object Rating Data type: Comma-Separated-Values
Brief description: Data Object rating patterns of EOL members in relation to their curator status.
File: oo_6177.csv Cynthia Parr, Nathan Wilson, Patrick Leary, Katja S. Schulz, Kristen Lans, Lisa Walley, Jennifer A. Hammock, Anthony Goddard, Jeremy Rice, Marie Studer, Jeffrey T. G. Holmes, Robert J. Corrigan, Jr.

## Figures and Tables

**Table 1.** Languages of common (vernacular) names.

Language	Common Names
English	690163
Spanish	114579
Chinese	87643
French	85973
German	69945
Japanese	51432
Portuguese	42497
Italian	39264
Czech	37455
Russian	35379
Danish	30775
Dutch	30775
Finnish	29785
Polish	24918
Other	280057

**Table 2.** Languages of text articles.

Language	Articles
English	3096313
Spanish	58978
Chinese	11678
Arabic	4807
Portuguese	2373
Dutch	1143
Indonesian	173
French	107
Other	10180

**Table 3.** **Subjects of text articles.** Combined topics include Wikipedia (n = 223571), Description (n = 49074), General Description (n = 45887), Brief Summary (n = 26862) and Biology (n = 8929). Subjects with fewer than 100 articles are not shown (Procedures, Legislation, Identification Resources, Systematics or Phylogenetics, Development).

Subject	Articles
Distribution	805503
Molecular Biology	434545
Combined Topics	354322
Type Information	326720
Habitat	292478
Conservation Status	144969
Threats	94140
Morphology	66571
Conservation	65618
Diagnostic Description	61512
Management	57894
Trends	57888
Size	55453
Description	49074
Associations	38677
Taxon Biology	26861
Uses	24458
Trophic Strategy	21563
Population Biology	17767
Taxonomy	16301
Ecology	15060
Reproduction	14996
Notes	14440
Migration	13991
Cyclicity	11880
Life Cycle	9759
Life Expectancy	8875
Behavior	6391
Key	6118
Diseases	4325
Use	4283
Evolution	2158
Risk Statement	2022
Look Alikes	1897
Dispersal	1649
Functional Adaptations	1438
Genetics	1000
Growth	785
Barcode	720
Education Resources	646
Physiology	269
Cytology	129
